# Filamentous Non-*albicans Candida* Species Adhere to *Candida albicans* and Benefit From Dual Biofilm Growth

**DOI:** 10.3389/fmicb.2019.01188

**Published:** 2019-05-31

**Authors:** Ruvini U. Pathirana, Andrew D. McCall, Hannah L. Norris, Mira Edgerton

**Affiliations:** Department of Oral Biology, School of Dental Medicine, University at Buffalo, Buffalo, NY, United States

**Keywords:** *Candida albicans*, non-*albicans Candida* species, biofilms, cell adhesion, hyphal formation, filamentation, cell detachment

## Abstract

Non-*albicans Candida* species (NACS) are often isolated along with *Candida albicans* in cases of oropharyngeal candidiasis. *C. albicans* readily forms biofilms in conjunction with other oral microbiota including both bacteria and yeast. Adhesion between species is important to the establishment of these mixed biofilms, but interactions between *C. albicans* and many NACS are not well-characterized. We adapted a real-time flow biofilm model to study adhesion interactions and biofilm establishment in *C. albicans* and NACS in mono- and co-culture. Out of five NACS studied, only the filamenting species *C. tropicalis* and *C. dubliniensis* were capable of adhesion with *C. albicans*, while *C. parapsilosis, C. lusitaniae, and C. krusei* were not. Over the early phase (0–4 h) of biofilm development, both mono- and co-culture followed similar kinetics of attachment and detachment events, indicating that initial biofilm formation is not influenced by inter-species interactions. However, the NACS showed a preference for inter-species cell-cell interactions with *C. albicans*, and at later time points (5–11 h) we found that dual-species interactions impacted biofilm surface coverage. Dual-species biofilms of *C. tropicalis* and *C. albicans* grew more slowly than *C. albicans* alone, but achieved higher surface coverage than *C. tropicalis* alone. Biofilms of *C. dubliniensis* with *C. albicans* increased surface coverage more rapidly than either species alone. We conclude that dual culture biofilm of *C. albicans* with *C. tropicalis* or *C. dubliniensis* offers a growth advantage for both NACS. Furthermore, the growth and maintenance, but not initial establishment, of dual-species biofilms is likely facilitated by interspecies cell-cell adherence.

## Introduction

Oropharyngeal candidiasis (OPC) is prevalent in populations of immunocompromised individuals, but treatment is hampered by limited drug options (Rautemaa and Ramage, 2011; Patel et al., 2012). Immunocompromised patients are at higher risk for subsequent systemic dissemination that may lead to mortality; for example individuals with oral mucositis as a result of cancer chemotherapy (Lalla et al., 2010). *Candida albicans* is by far the most predominant organism isolated from oral samples in OPC, however non-*albicans Candida* species (NACS) are frequent co-isolates (Patel et al., 2012). The prevalence of the opportunistic yeast *C. albicans* as the main causative agent of oral candidiasis is not surprising since up to 70% of healthy individuals are asymptomatic carriers as a part of their normal flora (Noble et al., 2016). However, more recent clinical reports using improved diagnostic methods based on molecular tools showed an abundance of other NACS during primary and recurrent infections (Pfaller and Diekema, 2007; Wu et al., 2017). *Candida dubliniensis* was an early NACS identified as a novel species in OPC from HIV infected patients (Sullivan et al., 1995); while *Candida glabrata, Candida tropicalis, Candida krusei, Candida lusitaniae, Candida parapsilosis, Candida kefyr, Candida guilliermondii*, and *Candida lipolytica* have been isolated from OPC patients including denture wearers, those with HIV infection, diabetes or other oral complications (Lalla et al., 2010; Rautemaa and Ramage, 2011; Williams and Lewis, 2011; Patel et al., 2012; Wu et al., 2017).

*Candida albicans* readily forms biofilms within the complex milieu of the human microbiome on mucosal surfaces and in-dwelling medical devices (Kojic and Darouiche, 2004; Kumar and Menon, 2006; Alnuaimi et al., 2013). Owing to the heterogeneity of microbial species in human normal flora, clinically relevant biofilms are mostly polymicrobial comprising different species within same genus as well as cross-kingdom microorganisms (Donlan and Costerton, 2002). The importance of interspecies interactions in colonization, disease progression, host response and drug resistance have been reviewed widely (Harriott and Noverr, 2011; Vipulanandan et al., 2018). *Candida* biofilms exhibit significantly higher drug resistance due to inherent drug resistance mechanisms [induction of efflux pumps and multidrug resistance (MDR) genes] and architectural composition of biofilms (extracellular matrix and subpopulations of persister cells) (Taff et al., 2013). Most clinical isolates of *C. albicans* as well as NACS show inherent drug resistance, thus mixed biofilms of *C. albicans* and NACS cause a more potent threat as well as being more difficult to eradicate (Ford et al., 2015; Whaley et al., 2016; Berkow and Lockhart, 2017). Further, it is hypothesized that oral carriage and persistence of NACS species may be promoted by the presence of *C. albicans* since pathogenic *Candida* species may have mutualistic or synergistic relationships that favor cohabitation (Shirtliff et al., 2009) leading to establishment of disease. In mixed biofilms, cell-cell adherence or co-aggregation play important roles in spatiotemporal biofilm development (Katharios-Lanwermeyer et al., 2014; Tati et al., 2016). The importance of co-aggregation during polymicrobial biofilms is well-studied for common oral pathogens (Shirtliff et al., 2009; Morales and Hogan, 2010; Diaz et al., 2014). Among those, *Staphylococcus aureus* (Harriott and Noverr, 2011), *Actinomyces* sp. (Grimaudo et al., 1996), *Streptococci gordonii* (Bamford et al., 2009), *Streptococcus mutans* (Metwalli et al., 2013; Falsetta et al., 2014), and *Fusobacterium* sp. (Hsu et al., 1990; Wu et al., 2015) have been reported to cohabit with *C. albicans*.

However, inter-species interactions between evolutionary close NACS with *C. albicans* during biofilm formation are still little known. We have shown previously that *C. albicans* serves as a scaffold upon which *C. glabrata* adheres (Tati et al., 2016). Among other NACS, the non-filamentous *C. glabrata* is phylogenetically more closely related to *Saccharomyces cerevisiae* than *C. albicans*, yet it has evolved to infect humans through its own adaptive mechanisms (Roetzer et al., 2011). *Candida albicans* responds to a wide variety of stimuli and possesses robust filamentation machinery enabling cells to form abundant biofilms (Noble et al., 2016). However, among the NACS, only *C. dubliniensis* and *C. tropicalis* produce true hyphae but have different requirements for filamentation, leading to different degrees of biofilm formation (Lackey et al., 2013). In spite of numerous studies of biofilm formation of NACS and *C. albicans*, including our own study of *C. glabrata* and *C. albicans* (Tati et al., 2016), greater detail in our understanding of these interspecies interactions is still required. The objective of this study is to determine development characteristics of NACS in mono- and co-culture with *C. albicans*, under flow induced biofilm formation conditions that model the oral cavity. Here we used a flow apparatus containing an ibidi^®^ μ-slide™ flow chamber which allows real-time imaging during biofilm development (McCall and Edgerton, 2017). This real time monitoring of biofilm allowed study of the kinetics of biofilm formation, thus identifying the critical steps in establishing a polymicrobial biofilm under flow dynamics.

## Materials and Methods

### Strains and Culture Media

Fluorescently labeled *Candida albicans* CAF2.1-dTom-NATr strain (CAF2-dTomato) (Gratacap et al., 2013) (kindly provided by Dr. Robert Wheeler, University of Maine) and *C. albicans* CAF2-1 (Fonzi and Irwin, 1993) (Δ*ura3::imm434/URA3*) were used as WT control. NACS strains used were *C. dubliniensis* Wü284 (O'Connor et al., 2010) (kindly provided by Dr. Kenneth Nickerson, University of Nebraska-Lincoln)*, C. tropicalis, C. parapsilosis* CLIB214 (kindly provided by Dr. Laura Rusche, University at Buffalo); *C. krusei* AR0397 and *C. lusitaniae* AR0398 were obtained from the antimicrobial resistance bank of the Centers for Disease Control and Prevention (CDC). All strains were routinely grown in YPD broth medium (YPD; Difco, Detroit, MI, USA) at 30°C with shaking at 220 rpm. For biofilm experiments, liquid Spider medium (pH 7.2) prepared as described previously (Liu et al., 1994), and RPMI 1640 supplemented with L-glutamine (Corning^®^ Cellgro) and 50 mg/ml of uridine (Sigma-Aldrich), were used.

### Cell-Cell Adherence

Cell-cell adherence was assayed as described previously (Tati et al., 2016). In brief, the stationary phase cell cultures of *C. albicans* CAF2-dTomato strain (Gratacap et al., 2013) were diluted to OD_600_ = 0.3 in 0.67% YNB broth (MP Biomedicals LLC.) supplemented with 1.25% N-Acetyl glucosamine (GlcNAc), and incubated for 4 h at 37°C to induce filamentation in mono-culture. For NACS strains, stationary phase cells were incubated in YNB supplemented with 1.25% glucose instead of GlcNAc. Cells were harvested by centrifugation, washed once in 1X PBS, and then resuspended in 1X PBS. Each NACS were then co-incubated with CAF2-dTomato in 1:1 ratio in PBS buffer for 60 min with gentle shaking and observed through Zeiss AxioObserver Z1 inverted fluorescence microscope (Carl Zeiss, Germany) using chambered slides [18 × 18 mm #1.5 cover glass (Fisher Scientific, Hampton, NH, USA), affixed on top of #1.5 cover glass spacers cut to 18 × 6 mm, and adhered to 25 × 75 × 1 mm glass microscope slides (Globe Scientific Inc., Mahwah, NJ, USA) with the non-toxic sealant Valap (Cold Spring Harbor Laboratory Press, 2015)].

### Static Biofilm Assays

For static biofilms, standard biofilm growth assay was used as described previously using 12 well-flat bottom plates (BD Falcon #353225) (Lohse et al., 2017). Overnight grown yeast cultures at stationary phase were diluted to either 1 × 10^6^ for mono-species, or 5 × 10^5^ cells/ml for dual-species, in 1 ml of Spider or RPMI media and plates were shaken at 100 rpm for 90 min at 37°C. Media supernatant was aspirated, and wells were washed twice with 1 ml of PBS buffer, followed by the addition of 1 ml of fresh media. Biofilms were grown in 12-well-polystyrene plates in Spider or RPMI media for 24 h under same incubation conditions; then cells were removed from wells and dehydrated to measure dry weight of biofilm from each well.

### Flow Induced Biofilm Quantitation

A flow apparatus previously described (McCall and Edgerton, 2017) was used to visualize and quantitate biofilm formation and cell to cell adhesion between species. For monoculture experiments, 1 × 10^6^ cells/ml of inoculum was used in a volume of 100 ml Spider media to seed the ibidi^®^ μ-slide™ (ibidi GmbH, Germany) flow chamber for 1 h (adhesion/attachment phase) at 37°C. The fluid (culture media) flow rate was maintained at 3.3 ml/min which generates a shear force of 0.8 dynes/cm^2^, equivalent to the shear force generated by saliva in the human oral cavity (Prakobphol et al., 1995). After 1 h, fresh sterile Spider medium was circulated through the system during the biofilm growth phase, and was passed through four sequential filters (20, 10, 2, and 0.22 μm) to ensure sterility of the cell-free media and used for recirculation across the slide flow chamber. Time-lapse darkfield images of biofilms were captured every 2 min during the 1 h attachment phase; and every 15 min during the growth phase. For dual species biofilm experiments, time-lapse images were recorded every 30 min using fluorescent and dark field channels in a Zeiss AxioObserver Z1 inverted fluorescence microscope (Carl Zeiss, Germany) housed in a XLmulti S1 incubator for the AxioObserver that allows efficient control of temperature at 37°C.

All image analyses were performed in the ImageJ software after conversion to an 8-bit grayscale file format as described in detail previously (McCall and Edgerton, 2017). Parameters of biofilm mass, coverage area, cell attachment, and normalized cell detachment were measured within the imaging region through densitometry analysis using the original images as previously described (McCall and Edgerton, 2017). Statistical analyses were performed in Graphpad Prism^®^ version 5.03 software. The percent of associated microcolonies in dual-species biofilms were counted manually by considering dual-species cell assembles as one unit (one associated colony) as a percent of total NACS cells presented in a single microscopic field for each experiment.

## Results

### Filamenting *Candida* Species Preferentially Adhere to Each Other

**Figure 1:**
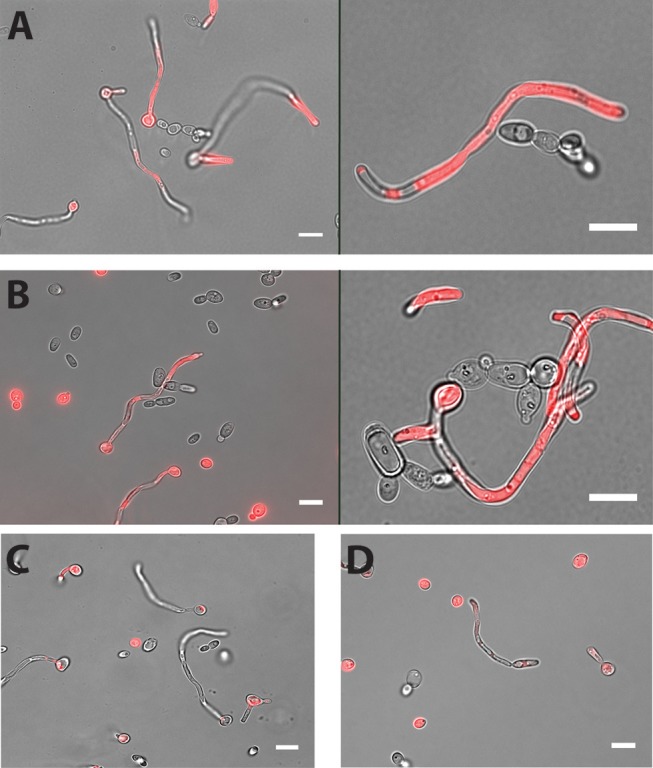
Both *C. dubliniensis* and *C. tropicalis* adhere to *C. albicans* hyphae. Hyphal cells of *C. albicans* were incubated with either *C. dubliniensis* or *C. tropicalis* cells at 1:1 ratio for 60 min in PBS buffer with mild agitation at 37°C. Adhesion of NACS along the length of *C. albicans* CAF2-dTomato was detected under florescence microscopy. *C. dubliniensis* **(A)** and *C. tropicalis* **(B)** both adhere to *C. albicans* hyphae but neither *C. krusei* **(C)** or *C. lusitaniae* **(D)** showed any adherence to *C. albicans* hyphae. Scale bar denotes 10 μm.

Cell-cell adhesion is one of the key steps in developing multi-species biofilms (Katharios-Lanwermeyer et al., 2014). To identify such interactions between *C. albicans* and NACS, we co-incubated *C. albicans* hyphae with five different NACS at a 1:1 cell ratio for 60 min in PBS buffer and observed their association by fluorescence microscopy using chamber slides. The use of chamber slides allowed us to observe cells in space without disturbing their natural arrangements in contrast to conventional wet mounts. We observed that both *C. dubliniensis* (Figure 1A) and *C. tropicalis* (Figure 1B) yeast cells adhered along *C. albicans* hyphae. Approximately one to two *C. dubliniensis* cells were found adhere to one *C. albicans* hyphae (average of 1.5 cells/hyphae) while one to three *C. tropicalis* cells were found adhere to one *C. albicans* hyphae (average of 2 cells / hyphae) per microscopic field (*n* ≥ 30). This interaction required the presence of *C. albicans* hyphae, since there was no adherence of *C. albicans* yeast with either *C. dubliniensis* or *C. tropicalis* yeasts cells (Supplemental Figure 1). In contrast, other NACS tested that included *C. krusei* AR0397 (Figure 1C), *C. lusitaniae* AR0398 (Figure 1D), and *C. parapsilosis* CLIB214 (not pictured) had no attachment to *C. albicans* hyphae. None of these species, (all unable to form hyphae), showed any interspecies cell-cell adherence and remained as isolated yeasts upon co-incubation.

### Spider Medium Supports Both Filamentation and Biofilm Growth in *C. albicans, C. dubliniensis*, and *C. tropicalis*

**Figure 2:**
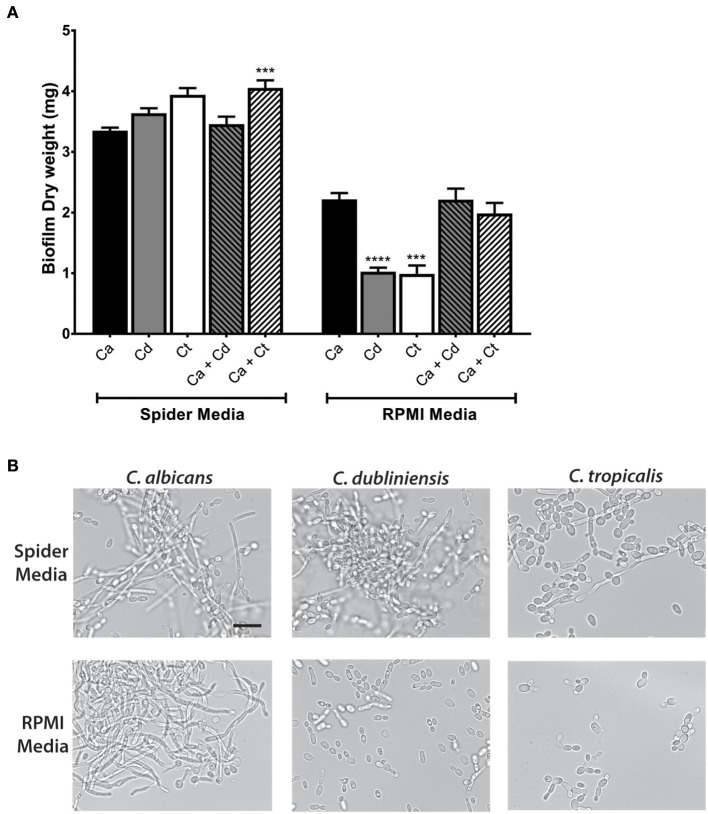
Spider media support abundant filamentation and biofilm formation in *C. albicans, C. dubliniensis* and *C. tropicalis*. **(A)** Biofilms were allowed to form under static conditions in Spider and RPMI media at 37°C with agitation at 100 rpm. The dry weights of the biofilms were measured after 24 h of growth. The increased biomass in Spider media but not in RPMI media indicates that Spider media supports growth of *C. albicans, C. dubliniesnsis* and *C. tropicalis* as single species biofilms to similar extent at 37°C in air when compared to RPMI medium. Ca, *C. albicans*; Cd, *C. dubliniensis*; Ct, *C. tropicalis*. **(B)** Yeast cells were incubated in liquid Spider or RPMI media at 37°C with agitation at 220 rpm and photographed after 6 h of growth. All three species were able to form filaments in Spider but not in RPMI media except *C. albicans*. Scale bar denotes 20 μm. ^***^P ≤ 0.001, ^****^P ≤ 0.0001.

Further study of dual species biofilms required a culture medium that supported equivalent growth, hyphae formation, and biofilm formation of all three species (*C. albicans, C. dubliniensis*, and *C. tropicalis*), so that differential growth or filamentation would not provide competitive advantage for any one species. Both Spider media and RPMI1640 have been widely used as biofilm growth media in previous *C. albicans* studies, therefore we tested single and dual biofilm growth under static conditions using Spider media or RPMI media. Dry weight measurements showed that Spider media supported equal biofilm growth of *C. albicans, C. dubliniensis*, and *C. tropicalis*; and the *C. albicans* and *C. tropicalis* dual species biofilm showed a significant growth increase with compared to *C. albicans* alone (^***^p < 0.001). In RPMI media, *C. dubliniensis* and *C. tropicalis* had significantly (^***^p < 0.001) reduced growth compared with *C. albicans* (Figure 2A), and neither dual species biofilm was significantly higher compared to that of *C. albicans* alone.

Microscopic observations of cells from each biofilm confirmed the presence of hypha in all three types of mono- and dual-species biofilms in Spider media. Since the degree of filamentation differs due to their inherent filamentation capacities [*C. tropicalis* is a poor filament former (Lackey et al., 2013)], we next performed filamentation assays by incubating each species in Spider and RPMI media at 37°C with shaking at 220 rpm. Spider media induced robust filamentation in *C. albicans* and *C. dubliniensis*, and readily apparent filamentation in *C. tropicalis* (Figure 2B) as expected. However, while RPMI supported hyphae formation in *C. albicans*, filamentation did not occur for either *C. dubliniensis* or *C. tropicalis* when cultured in RPMI media (Figure 2B) [this study and (O'Connor et al., 2010; Galán-Ladero et al., 2013)]. Therefore, we chose to use Spider media for further study of inter-species growth in our flow system since this media supported both growth (Figure 2A) and filamentation (Figure 2B) of all three *Candida* species.

### Detachment Is a Key Determinant of Flow Induced Mono-Culture Biofilms

**Figure 3:**
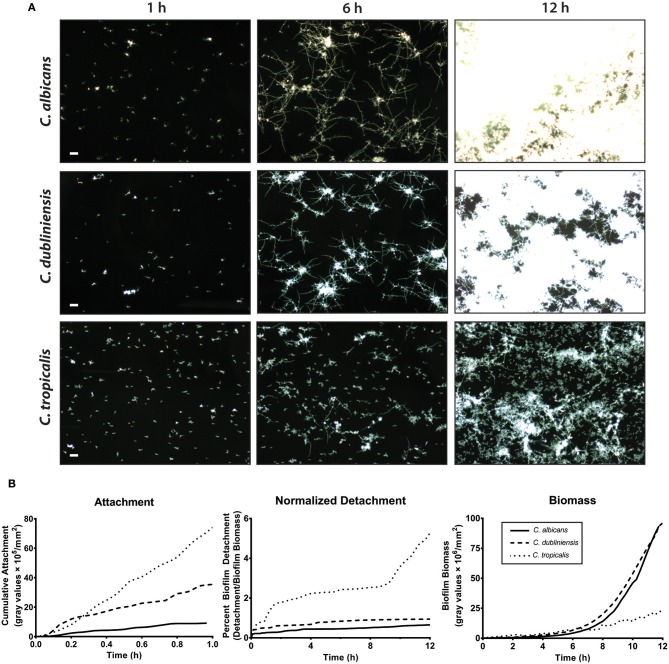
*C. albicans* and *C. dubliniensis* made abundant biofilms when compared to *C. tropicalis* under flow dynamics. To grow biofilms under flow (flow rate of 3.3 ml/min), the ibidi^®^ flow chamber was seeded with 1 × 10^5^ cells/ml for 1 h (adhesion phase) and then fresh media circulated throughout the growth phase (*ca*.18 h). **(A)** Representative dark field images of biofilm formation under flow are shown for *C. albicans* (top row), *C. dubliniensis* (middle row), and *C. tropicalis* (bottom row) at 1, 6, and 12 h of growth. The scale bar indicates 50 μm. **(B)** The rate of cell attachment, the detachment rate normalized to the biomass over time, and the change in biomass over time, within the imaging region are shown for *C. albicans* (solid line), *C. dubliniensis* (dashed line) and *C. tropicalis* (dotted line) as determined by densitometry analysis on ImageJ software. Data are means of *n* ≥ 3 experiments.

We first compared the ability of mono-cultures of *C. albicans, C. dubliniensis* and *C. tropicalis* to form biofilms under flow dynamics in Spider media. The flow system used here enabled us to distinguish both cell adhesion and growth phases of biofilm formation as described previously (McCall and Edgerton, 2017). During the adhesion phase, the ibidi^®^ flow chamber was seeded with 1 × 10^5^ cells/ml for 1 h. The parameters of inoculum size and adhesion time were chosen to allow sufficient seeding for all three species of *Candida* used in this study, therefore avoiding over- or under- growth in Spider media. The second phase (biofilm growth phase) occurred over the next 16–18 h during which only fresh culture media was circulated through the ibidi^®^ chamber under the same flow rate. Representative images were chosen for three different time points (1 h, 6 h and 12 h) during the course of biofilm formation, shown in Figure 3A and Supplemental Videos S1–S3.

The cell attachment rate was quantified as a percent of covered area with attached cells to the total area during the attachment phase. The cumulative attachment of cells to the substratum during the first hour showed approximately an 8-fold increase for *C. tropicalis* and a 4-fold increase for *C. dubliniensis* compared to *C. albicans* (Figure 3B). By the end of the attachment phase, all adherent cells of both *C. albicans* and *C. dubliniensis* started to develop germ tubes (Supplemental Videos S1–S3). Once the growth phase started, adhered cells rapidly extended their germ tubes radially, forming microcolonies as we have described previously (McCall and Edgerton, 2017) but with more robust filamentation largely due to the filament promoting Spider medium. *Candida albicans* extended filaments began forming blastospores after 7–8 h of growth; however this blastopore formation was comparatively minimal in *C. dubliniensis*. *C. tropicalis* was able to form true hyphae (Figure 2B); however, we did not observe germ tube formation for most adherent *C. tropicalis* cells. Only about 20–30% of adherent *C. tropicalis* cells initiated filamentation while the remainder stayed in yeast form. These remaining yeasts continued to grow by budding and these budding cells made clumps of yeast cells, which eventually detached either completely or partially during the growth phase (Supplemental Videos S1–S3). Thus, the normalized detachment rate (proportion of detached cells compared to total biomass) was significantly higher for *C. tropicalis* (8-fold over the first hour) compared to either *C. albicans* or *C. dubliniensis* that had a similar detachment rate to each other (Figure 3B). This resulted in a low biomass level for *C. tropicalis*, and these cells did not show the exponential increase in biomass that occurred around 7 h of growth that was found for *C. albicans* and *C. dubliniensis* (Figure 3B). By 12 h of growth, the architecture of the mature biofilm was similar for *C. albicans* and *C. dubliniensis*, in that both produced thick biofilms with extensive filamentation having similar total biomass (Figure 3B). *C. tropicalis* formed comparatively thin biofilms, so that even by 12 h the biofilm consisted mainly of yeast cells with some filamentation. The higher detachment rate in *C. tropicalis*, combined with poor filamentation, resulted in a reduced total biomass even though this species had the highest initial cell attachment compared with the other two species.

### Mosaics of Co-species Interactions Are Observed in Dual-Species Flow Induced Biofilms

**Figure 4:**
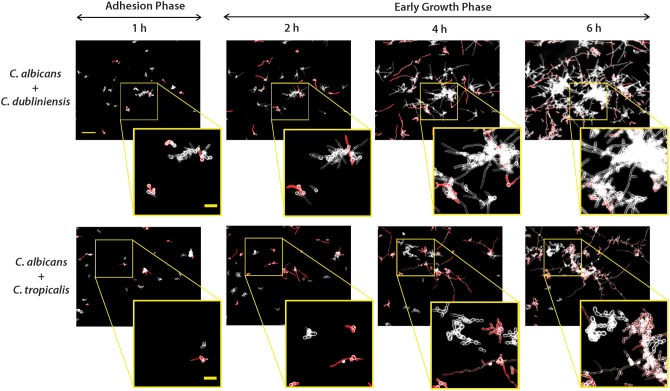
Dual-species biofilm formation showed species-species interactions during early biofilm growth under flow. Biofilms were grown similarly to mono-culture, except inoculum of 1 × 10^5^ cells/ml for each species was used for dual-culture experiments. Representative fluorescence and dark field merged images of biofilm formation under flow are shown for *C. albicans* CAF-2 tdTomato (red fluorescence) co-inoculated with *C. dubliniensis* or *C. tropicalis* at 1, 2, 4, and 6 h of growth. The scale bar indicates 50 μm. To visualize co-species interactions, a representative area from the same location was enlarged and shown in squared images where the scale bar indicates 20 μm.

To determine the interspecies development characteristics of NACS during co-culture with *C. albicans*, we then grew each NACS with *C. albicans* CAF2-dTomato strain to identify whether interspecies interactions enhance biofilm formation. Fluorescent images were merged with dark field images to enhance and distinguish between the two species, so that fluorescent *C. albicans* appeared red and non-fluorescent NACS were visualized as white outlines of the cell wall (Figure 4 and Supplemental Videos S4, S5). In an initial experiment, we confirmed that filamentation of the labeled *C. albicans* CAF2-dTomato strain was similar to that of unlabeled *C. albicans* CAF2-1 (Supplemental Figure 2). Previous animal model studies conducted using the same CAF2-dTomato strain showed virulence properties this strain were similar to those of its unlabeled parental strain (Brothers et al., 2013; Chen et al., 2015) indicating labeling did not affect its phenotypic or virulence properties.

As for monocultures, *C. albicans* formed germ tubes by the end of the 1 h adhesion period, and both dual species pairs [*C. albicans* and *C. dubliniensis* (Ca+Cd); *C. albicans* and *C. tropicalis* (Ca+Ct)] showed distinct cell to cell adhesion (Figure 4). During the first hour, clusters of *C. dubliniensis* yeast cells were observed to attach to *C. albicans* hyphae, and by 2 h clusters of *C. dubliniensis* attached to *C. albicans* formed hyphae that extended along the length of *C. albicans* filaments. Over the next 6 h of growth, these filamented *C. dubliniensis* cell clusters remained attached to *C. albicans* and continued to grow outward from these anchorage points (Figure 4). In contrast, *C. tropicalis* formed budded cell clumps that attached more sparsely to *C. albicans* hyphae during the first 2 h of co-culture with flow. Many *C. tropicalis* cell clusters were adherent to the substrate independently from *C. albicans*, and appeared to grow more slowly in co-culture even when attached to *C. albicans* hyphae. Surprisingly, *C. tropicalis* did not filament in the presence of *C. albicans* at any time point during 10 h of co-culture, despite forming hyphae in monoculture (Supplemental Figure 3); suggesting that *C. tropicalis* hyphal formation is repressed by *C. albicans*.

**Figure 5:**
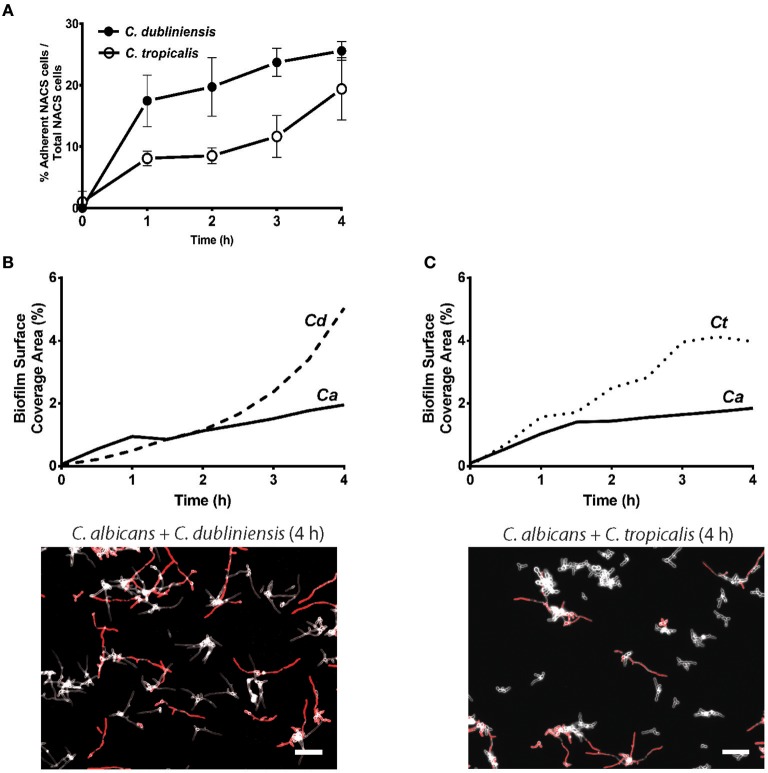
NACS predominate over *C. albicans* hyphae during early biofilm formation. **(A)** The percent of associated colonies in dual-species biofilm over the first 4 h was counted manually by considering NACS + *C. albicans* cell assemblies as one unit (one associated colony) as a percent of total NACS cells present. The increase of dual species associated colonies was indicative of a preference for inter-species cell-cell adherence. Quantitative analysis of dual-species biofilm formation using surface coverage area of individual species showed *C. dubliniensis* **(B)** and *C. tropicalis* **(C)** grow rapidly than *C. albicans* hyphae. Two representative images at 4 h time frames were shown beneath each graph. Scale bar denotes 50 μm. Ca, *C. albicans*; Cd, *C. dubliniensis*; Ct, *C. tropicalis*.

**Figure 6:**
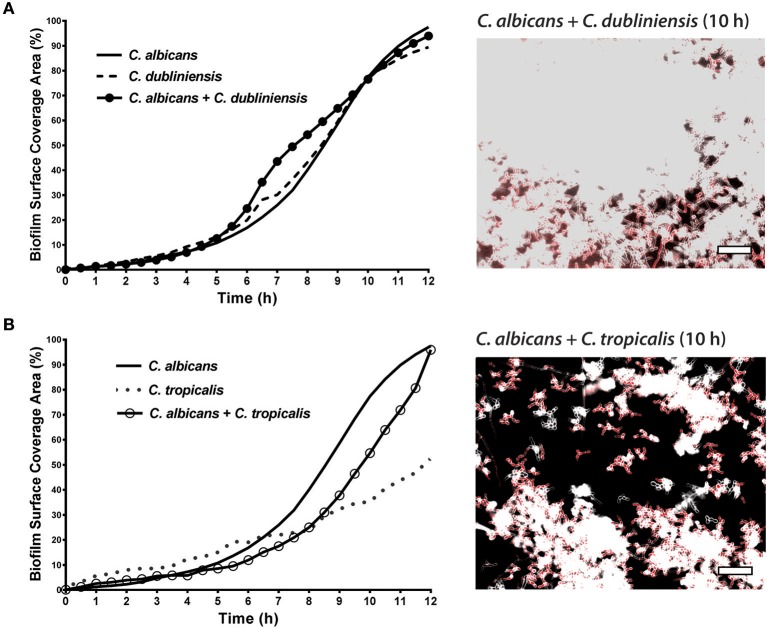
In dual species biofilms, abundance and hyphal development of each species is affected by each cohabiting species' growth characteristics. The biofilm surface coverage areas were calculated by the threshold pixel intensities on ImageJ software, which reflects the growth rate of the biofilms. **(A)** *C. albicans* + *C. dubliniensis* dual species achieve more rapid biofilm surface coverage together but reach the same biofilm coverage area by 12 h. **(B)** *C. albicans* + *C. tropicalis* dual species showed less surface coverage when compared to *C. albicans* alone but more coverage with compared to *C. tropicalis* alone. Representative images of *C. albicans* + *C. dubliniensis* and *C. albicans* + *C. tropicalis* dual species biofilms are shown at 10 h. The higher cell densities are reflected by the confluent cell growth. Scale bar denotes 50 μm.

To quantitate the levels of co-adhesion of each NACS, we measured the percentage of each species that were adherent to *C. albicans* over 4 h (Figure 5A). About 2-fold more *C. dubliniensis* cell groups than *C. tropicalis* were found to adhere to *C. albicans* over the initial 4 h examined (after 4 h the higher cell density did not allow quantitation to enable distinguishing the two species). By 4 h of dual species growth, both *C. dubliniensis* and *C. tropicalis* had higher biofilm surface coverage area than *C. albicans*—a reflection of the higher density of cell clusters of adherent NACS to *C. albicans* hyphae (Figures 5B,C). However, we expected that this co-adhesion would improve formation of the total dual-species biofilm. To compare single and dual-species biofilms, surface coverage area for these pairs was measured over 12 h to confluence (Figure 6). As we had previously found for total biomass (Figure 1), biofilm surface coverage area for *C. dubliniensis* alone was equivalent to *C. albicans* alone, likely a reflection of similar levels of attachment, detachment, and filamentation. However, dual species Ca+Cd biofilm surface coverage was higher over 5–10 h of growth, after which both mono and dual species had similar coverage areas. Thus, Ca+Cd dual-species achieve more rapid biofilm surface coverage together, and ultimately attain confluence as in mono-culture given sufficient time of growth (Figure 6A). In contrast, *C. tropicalis* alone had higher biofilm surface coverage than *C. albicans* up to 6 h, due to its higher surface attachment rate (Figure 6B); however mono-cultures of *C. tropicalis* did not have the exponential increase in biofilm surface area displayed by both *C. albicans* or *C. dubliniensis* after 6 h. Interestingly, Ca+Ct dual species had similar biofilm surface coverage as *C. albicans* alone up to 5 h, however Ca+Ct biofilm coverage lagged compared to *C. albicans* alone from 5 to 11 h, although the coverage was substantially higher than for *C. tropicalis* alone (Figure 6). Overall, Ca+Ct dual species biofilms had slower biofilm coverage than either *C. albicans* or *C. dubliniensis* alone; however Ca+Ct dual species also ultimately grew to confluence, unlike *C. tropicalis* that only had 50% biofilm surface coverage area even after 12 h growth.

## Discussion

Since standard static biofilm assays do not clearly distinguish inter-species interactions in real time (Bamford et al., 2009; Tati et al., 2016; Weerasekera et al., 2016), we adapted a flow model (McCall and Edgerton, 2017) to quantify dual-species biofilm development. In examining five NACS for their ability to adhere to *C. albicans* hyphae, we found that only two species, *C. tropicalis* and *C. dubliniensis*, were capable of forming these associations. These two species have a close phylogenetic relationship to *C. albicans* and share the ability to form true hyphae; while *C. lusitaniae, C. parapsilosis*, and *C. krusei* are less closely related to *C. albicans*, and only can produce pseudohyphae (Lackey et al., 2013). Although we noted a significant increase in the dry weight of statically grown biofilms of *C. albicans* and *C. tropicalis* suggesting potential synergy between these species; these experiments were limited in their ability to study each species individually within mixed culture growth. For mixed species experiments, it was necessary to have equivalent hyphal production since the degree of filamentation for each species (*C. albicans, C. dubliniensis*, and *C. tropicalis*) was directly proportional to the biofilm biomass. Therefore, we employed Spider media to achieve similar levels of filamentation and biofilm growth for dual species quantitation. Furthermore, *C. albicans* biofilms grown in Spider media, which contains beef extract as the component that induces filamentation, are more susceptible to fluconazole treatment and leukocyte infiltration than biofilms formed in RPMI medium (Daniels et al., 2013).

As we previously found for *C. albicans* (McCall and Edgerton, 2017); this study also identified the normalized rate of detachment as the key determinant for mono-culture biofilm formation for *C. dubliniensis* and *C. tropicalis*. Surprisingly, both *C. dubliniensis* and *C. tropicalis* had a higher initial rate of attachment than *C. albicans* in mono-culture, given the perception that *C. albicans* is most adherent of the three. However, later events in biofilm growth modulated ultimate surface coverage. *C. dubliniensis* biofilm grew to confluence in a similar time frame to *C. albicans* in spite of its higher rate of initial attachment. Also in spite of the high initial attachment, we also found that *C. tropicalis* had a high rate of detachment from the substrate over 12 h, which prevented growth to the same level of biomass seen for *C. albicans* and *C. dubliniensis*. The differences in later adhesion maintenance between *Candida* spp. are apparent over time and impact biofilm formation. Hyphal-specific adhesins are likely candidates that are differentially expressed between these species. For example, *C. albicans* Als3 is a well-characterized hyphal-specific adhesin involved in biofilm formation and adhesion (Silverman et al., 2010; de Groot et al., 2013). Although *C. tropicalis* is a comparatively weak hyphae former (Lackey et al., 2013), it expresses similar adhesins including Als3 and is considered almost as adherent as *C. albicans* (Zuza-Alves et al., 2017). In contrast, although *C. albicans* and *C. dubliniensis* contain genomes which are 98% syntenic (Moran et al., 2012), *C. dubliniensis* does not express Als3 and its other surface proteins are highly dissimilar (Moran et al., 2012).

We found that the early phases of dual-species biofilm development followed similar kinetics of attachment and/or detachment events as for mono-culture biofilms. Even though both *C. dubliniensis* and *C. tropicalis* yeast cells adhere to *C. albicans* hyphae, this cell-cell adherence played a minor role in the attachment phase and in establishing the early time points (0–4 h) of these dual-species biofilms. However, over the first 4 h of biofilm growth, the percentage (compared to total cell numbers) of *C. dubliniensis* and *C. tropicalis* cells adhering to *C. albicans* increased, indicating a preference for interspecies cell-cell interactions. Additionally, up to 4 h, *C. dubliniensis* and *C. tropicalis* dominated over *C. albicans* based upon quantitation of biofilm surface coverage, which may be due to higher initial attachment. At later time points, the growth of each species was visibly influenced by interspecies interactions. Co-species biofilms of Ca+Cd increased surface coverage faster, while Ca+Ct was slower, than for biofilms of mono-culture *C. albicans*. *C. albicans* predominated over the growth of *C. dubliniensis* and *C. tropicalis* when the biofilms reached maturation, though neither species was eliminated. Therefore, growth and maintenance, but not initial establishment, of dual-species biofilms is likely facilitated by interspecies cell-cell adherence. Significantly, we found that hyphal production of *C. tropicalis* was suppressed when grown together with *C. albicans* despite being cultured in Spider media. This repression is likely due to production of quorum sensing molecule farnesol by *C. albicans*; and *C. tropicalis* is particularly sensitive to farnesol (Weber et al., 2010). Quorum sensing among fungal species is understudied and may have a substantial impact on biofilm growth of *Candida* species *in vivo*.

*Candida albicans* is the most frequently isolated *Candida* species found in studies of carriage in healthy individuals and multiple disease states, while *C. dubliniensis* and *C. tropicalis* are isolated less often (Patel et al., 2012; Ribeiro Ribeiro et al., 2015; Callejas-Negrete et al., 2016; Jain et al., 2016; Maheshwari et al., 2016; Mohammadi et al., 2016; Goulart et al., 2018; Norris et al., 2018). Patients with HIV/AIDS tend to harbor a high proportion of NACS; however, *C. albicans* usually remains the predominant species (Patel et al., 2012; Ribeiro Ribeiro et al., 2015; Callejas-Negrete et al., 2016; Maheshwari et al., 2016). A higher proportion of NACS has also been shown in diabetes mellitus patients compared to healthy controls (Mohammadi et al., 2016). In contrast, a study of individuals undergoing treatment for oral cancer found that *C. tropicalis* was the most common *Candida* isolate (Jain et al., 2016). *C. tropicalis* has also been identified as the most common cause of candidemia in cancer patients (Wu et al., 2017). Our findings support the view that *C. albicans* cells tend to predominate over NACS in mature biofilms in healthy individuals. However, disease states may modulate this balance—for example by the presence of blood products that promote filamentation in NACS that do not typically produce hyphae.

Our results suggest that there may be a benefit for both species in a co-colonized environment; the rapid establishment of a mature biofilm that occurs in Ca+Cd co-culture indicates a potential competitive advantage against other microbiota *in vivo*. We observed a lag in biofilm growth in Ca+Ct co-culture compared to Ca alone; however, surface coverage of the biofilm past 8.5 h ultimately exceeded what *C. tropicalis* was capable of producing alone, indicating a clear benefit for *C. tropicalis*. The potential advantages of rapidly establishing and participating in a mature biofilm are numerous, including protection from environmental stresses (Pemmaraju et al., 2016), antifungal agents (Chandra et al., 2001), and other competing microbiota (Deschaine et al., 2018). Conversely, mono-culture biofilms of *C. tropicalis* have also been shown to be more resistant to antifungal diffusion than *C. albicans* biofilms (Al-Fattani and Douglas, 2006), which could offer a benefit to *C. albicans* in Ca+Ct biofilms. Nonetheless, the outcome of interactions between *C. albicans* and NACS is highly dependent upon growth environment. A study by Kirkpatrick et al. (2000) discovered that *C. dubliniensis* is able to tolerate rigorous competitive pressure from *C. albicans* in a catheter biofilm model, while *C. albicans* dominates in planktonic co-culture (Kirkpatrick et al., 2000). An antagonistic relationship between *C. tropicalis* and *C. albicans* was reported in another study in which reduction of *C. albicans* filamentation and reduced expression of *C. albicans* virulence genes was found in the presence of *C. tropicalis* (de Barros et al., 2018). We also found antagonism, but in our dual species biofilms *C. tropicalis* filamentation was suppressed by *C. albicans*. However, in spite of this competition, *C. tropicalis* still garners an advantage in biofilm growth from association with *C. albicans*.

## Conclusions

This study offers insight into the way that inter-species interactions of *Candida* modify behavior and fitness in biofilms. We conclude that dual culture biofilm of *C. albicans* and *C. tropicalis* or *C. dubliniensis* offers a growth advantage for both NACS. We have identified the normalized rate of substrate detachment as the primary determinant for both mono- and dual-species biofilm formation. In addition, we found that both *C. tropicalis* and *C. dubliniensis*, in dual biofilm growth with *C. albicans*, show a preference for inter-species cell-cell associations so that populations of NACS are additionally maintained within mature dual species biofilms. Our results indicate that growth and maintenance, but not initial establishment, of dual-species biofilms are supported by interspecies adherence. Therefore, among filamenting NACS, dual species growth benefits both species in biofilm development.

## Data Availability

The raw data supporting the conclusions of this manuscript will be made available by the authors, without undue reservation, to any qualified researcher.

## Author Contributions

RP, AM, and ME conceived and designed the experiments. RP, AM, HN, and ME wrote the manuscript. RP, AM, and HN conducted the experiments and analyzed the flow biofilm data. RP conducted the other experiments.

### Conflict of Interest Statement

The authors declare that the research was conducted in the absence of any commercial or financial relationships that could be construed as a potential conflict of interest.
