# Supplementary material for: Filamentous Non-albicans Candida Species Adhere to Candida albicans and Benefit From Dual Biofilm Growth
Source: Front Microbiol. 2019 May 31;10:1188. doi: 10.3389/fmicb.2019.01188 (PMC6558389; doi:10.3389/fmicb.2019.01188)
Supplement: Supplementary file 6 [file Data_Sheet_1.pdf]

Supplementary Material

1 Supplementary Figures

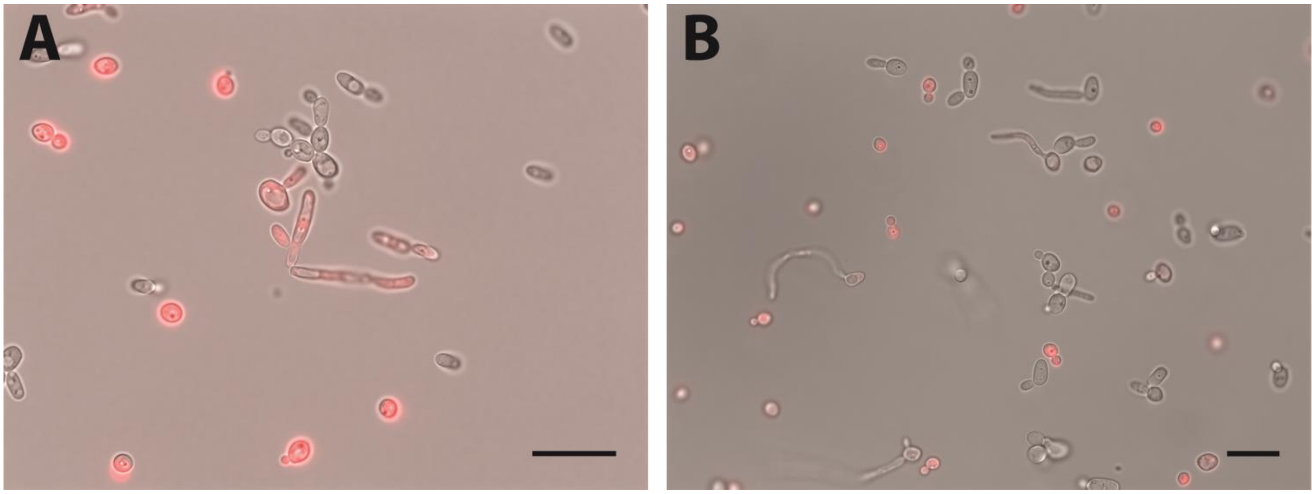

Supplemental Figure 1. *C. albicans* yeast morphotype cells show no adherence to *C. dubliniensis* or *C. tropicalis*. *C. albicans* cells supplemented in 1.25% N-Acetyl glucosamine (GlcNAc) were incubated with either *C. dubliniensis* or *C. tropicalis* cells at 1:1 ratio for 60 min in PBS buffer with shaking at 90 rpm at 37 ^{0}C. Adhesion of NACS to *C. albicans* CAF2-dTomato (red) was detected under florescence microscopy. *C. dubliniensis* (A) and *C. tropicalis* (B) do not adhere to *C. albicans* yeast morphotype. Scale bars = 18 microns.

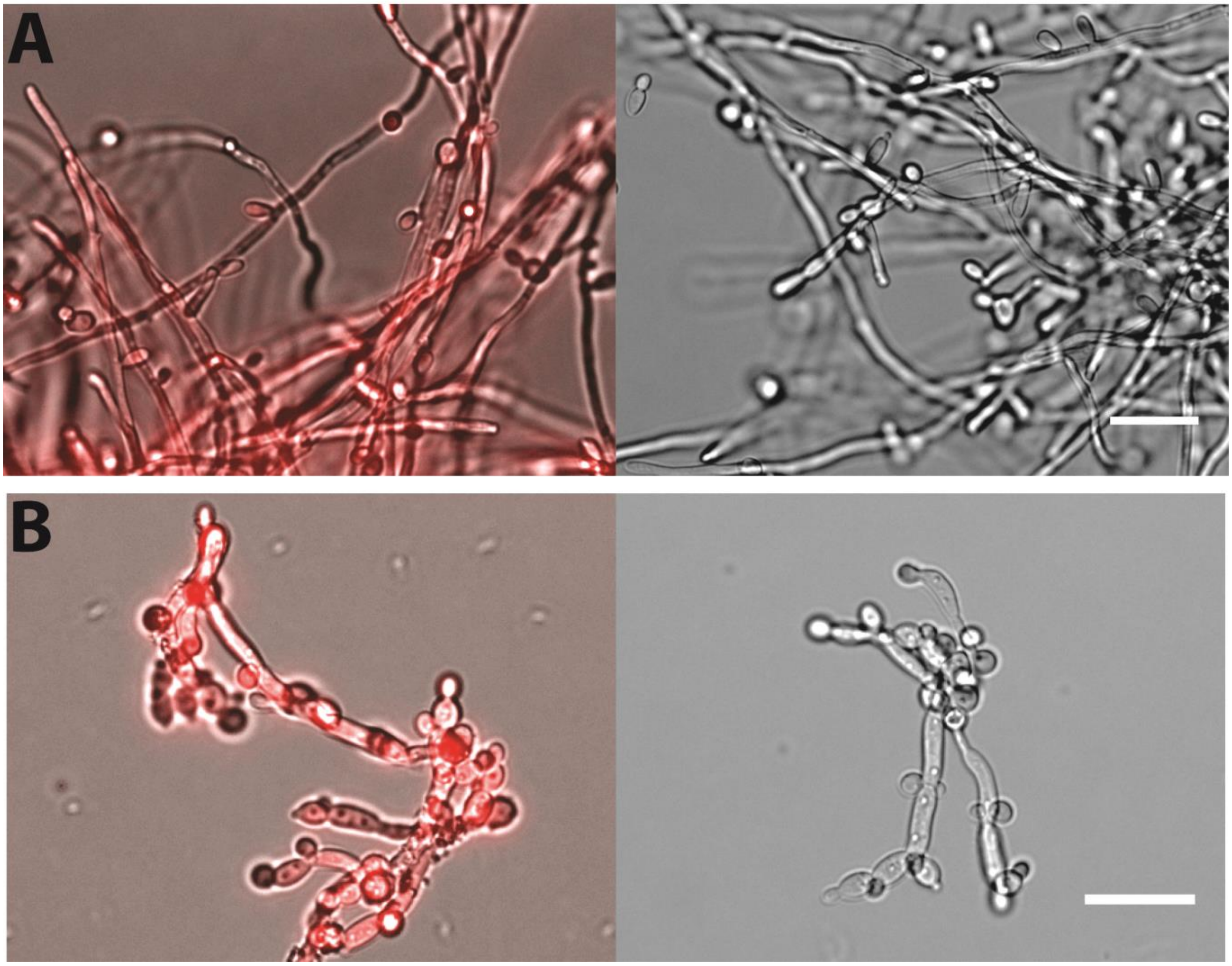

Supplemental Figure 2. Filamentation *C. albicans* fluorescent strain CAF2-dTomato is equivalent to its parental strain CAF2-1. Cells were grown for 6 h at 37°C at 220 rpm in Spider (A) or RPMI (B) media to induce filamentation. CAF2-dTomato cells (red) were imaged under fluorescence and bright field microscopy, and CAF2-1 cells were imaged with bright field only. Scale bars = 20 microns.

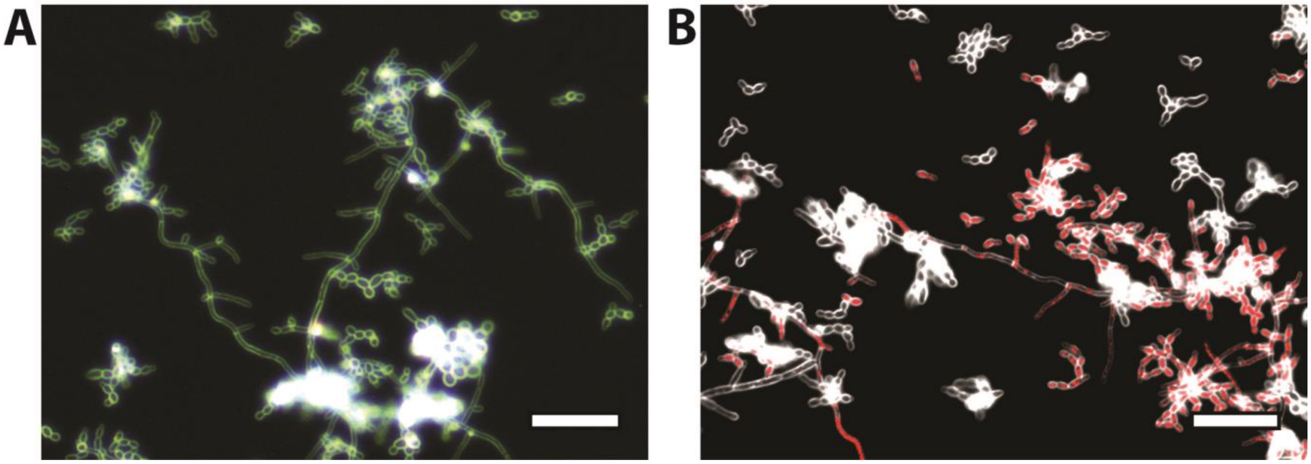

Supplemental Figure 3. *C. tropicalis* hyphal formation is repressed by the presence of *C. albicans*. (A) Representative image of *C. tropicalis* in mono-culture biofilm under flow at 7 h (B) Representative image of *C. tropicalis* in co-culture biofilm with *C. albicans* under flow at 7 h. Note that longer filaments are produced when growing *C. tropicalis* by itself and shorter filaments and cell clumps (white margined cells) appeared during co-culture with *C. albicans* (red cells) at the same time point. Scale bars = 50 microns.

2 Supplementary Video Descriptions

Supplemental Video 1. Mono-culture biofilm timelapse: *C. albicans*

Supplemental Video 2. Mono-culture biofilm timelapse: *C. dubliniensis*

Supplemental Video 3. Mono-culture biofilm timelapse: *C. tropicalis*

Supplemental Video 4. Dual-culture biofilm timelapse: *C. albicans* + *C. dubliniensis*

Supplemental Video 5. Dual-culture biofilm timelapse: *C. albicans* + *C. tropicalis*
